# Application of a target array Comparative Genomic Hybridization to prenatal diagnosis

**DOI:** 10.1186/1471-2350-11-102

**Published:** 2010-06-24

**Authors:** Ji Hyeon Park, Jung Hoon Woo, Sung Han Shim, Song-Ju Yang, Young Min Choi, Kap-Seok Yang, Dong Hyun Cha

**Affiliations:** 1 Department of Obstetrics and Gynecology, CHA Bundang Medical Center, CHA University, Seongnam-si, Korea; 2 Department of Bio Chip Service, Macrogen Inc., Seoul, Korea; 3 Genetic Laboratory, Fertility Center of CHA Gangnam Medical Center, CHA University, Seoul, Korea; 4 Department of of Obstetrics and Gynecology, Seoul National University College of Medicine, Seoul, Korea; 5 Department of Obstetrics and Gynecology, CHA Gangnam Medical Center, CHA University, Seoul, Republic of Korea, Seoul, Korea

## Abstract

**Background:**

While conventional G-banded karyotyping still remains a gold standard in prenatal genetic diagnoses, the widespread adoption of array Comparative Genomic Hybridization (array CGH) technology for postnatal genetic diagnoses has led to increasing interest in the use of this same technology for prenatal diagnosis. We have investigated the value of our own designed DNA chip as a prenatal diagnostic tool for detecting submicroscopic deletions/duplications and chromosome aneuploidies.

**Methods:**

We designed a target bacterial artificial chromosome (BAC)-based aCGH platform (MacArray™ M-chip), which specifically targets submicroscopic deletions/duplications for 26 known genetic syndromes of medical significance observed prenatally. To validate the DNA chip, we obtained genomic DNA from 132 reference materials generated from patients with 22 genetic diseases and 94 clinical amniocentesis samples obtained for karyotyping.

**Results:**

In the 132 reference materials, all known genomic alterations were successfully identified. In the 94 clinical samples that were also subjected to conventional karyotyping, three cases of balanced chromosomal aberrations were not detected by aCGH. However, we identified eight cases of microdeletions in the Yq11.23 chromosomal region that were not found by conventional karyotyping. This region harbors the DAZ gene, and deletions may lead to non-obstructive spermatogenesis.

**Conclusions:**

We have successfully designed and applied a BAC-based aCGH platform for prenatal diagnosis. This platform can be used in conjunction with conventional karyotyping and will provide rapid and accurate diagnoses for the targeted genomic regions while eliminating the need to interpret clinically-uncertain genomic regions.

## Background

Speed and precision are two major requirements in prenatal chromosome analyses. Conventional G-banded karyotyping remains the gold standard in prenatal genetic diagnosis, but it is time-consuming and labor-intensive. Routinely, about 10-14 days is required to obtain the result and this may increase the patient's anxiety. To overcome these limitations, rapid fluorescent in situ hybridization (FISH), quantitative fluorescent polymerase chain reaction (QF-PCR), and multiplex ligation-dependent probe amplification (MLPA) have been developed and are widely used as adjuncts to conventional methods for detecting common chromosome aneuploidies such as trisomies 21, 13, 18, X and Y [1-6]. However, only a few loci may be tested at a time, so all those methods can usually be performed only in a limited manner based on phenotype.

In addition, conventional G-banded karyotyping can hardly detect small deletions and duplications that result in serious clinical conditions such as congenital anomalies, mental retardation and developmental delay during the fetal stage as well as after birth. Recently, array comparative genomic hybridization (aCGH) has been used in prenatal as well as postnatal clinical cytogenetics to detect submicroscopic chromosomal imbalances [7-9]. This technique gives rapid results and multiplex detection of both numerical and unbalanced structural abnormalities with much higher resolution and wider coverage than conventional karyotyping and other molecular cytogenetic techniques. Several types of platform with different resolutions for aCGH have been developed. More recently, genome-wide oligonucleotide aCGH chips with several-kb resolutions have become commercially available. However, for clinical purposes, especially prenatal diagnosis, these high-resolution whole-genome aCGH chips are not adequate because of difficulties of interpretation mainly resulting from benign copy number variations (CNVs), hybridization quality and cost. On the other hand, targeted bacterial artificial chromosome (BAC)-based aCGH methods have been successfully used in prenatal diagnosis [7,10-12].

In this study, we have developed and evaluated a low density BAC-array prenatal DNA chip targeted to 26 known genetic syndromes of medical significance, caused by 19 microdeletions/duplications as well as numerical changes in chromosomes 13, 18, 21, X and Y.

## Methods

### Subjects

To validate the array, because we could not obtain four microdeletion syndromes - Sotos syndrome, monosomy 1p36, SRY region of Yp and Kallmann syndrome - we used 132 reference materials of 22 genetic disorders, 15 for microdeletions or duplications and 7 for chromosome aneuploidies, as positive controls. The cell lines were purchased from the Coriell Cell Resource http://ccr.coriell.org/nigms/products/pdr.html.

For clinical application of the array, all protocols were reviewed and approved by the Internal Review Board of CHA General Hospital and written informed consent was received from all participants. In total, 94 clinical samples were admitted. Sample types and reasons for referral are summarized in Table 1. During September 2007 through July 2008, amniocenteses were performed at the 15^th ^to 20^th ^weeks of gestation on 94 pregnant women who visited CHA General Hospital, Korea. The mean age of the pregnant women was 34.4 years.

**Table 1:** Indications for chromosome analyses in clinical cases admitted

Sample type	Indications*	No. of samples
Amniotic fluid	Advanced maternal age	42
	Abnormal serum screening results	38
	Abnormal ultrasonogram	12
	Family history of genomic disorder or cytogenetic abnormalities	2
	Pregnancy after artificial reproductive technology (intracytoplasmic sperm injection)	6
	Rubella IgM positive	1
	Others	3

Total		94

Some patients had two or more indications

For all positive controls and clinical samples, experiments were performed blind to exclude biases. Cytogenetic analyses and aCGH were independently executed in two different laboratories.

### Cytogenetic analysis

Amniocytes were grown in Chang Medium^® ^in situ (Irvine Scientific, Santa Ana, CA) and in Bio-AMF-2 complete medium (Biological Industries, Israel), with 5% CO_2 _in a 37°C incubator. All procedures including in situ culture, harvesting, slide preparation and GTG-banding followed routine protocols [13]. For each sample, more than 20 metaphases were examined.

### Manufacture of BAC-mediated array CGH, MacArray™ M-chip

The MacArray™ M-chip was developed to detect the 19 microdeletion syndromes as well as numerical aberrations (i.e. aneuploidies) in chromosomes 13, 18, 21, X and Y. A total of 181 BAC clones were prepared from our DNA BAC libraries (Macrogen, Korea) to design overlapping probes that detected consecutive and consistent alterations of the genome. All selected clones were two-end sequenced using an ABI PRISM 3700 DNA Analyzer (Applied Biosystems, USA), and their sequences were blasted and mapped according to their positions. The locus specificities of selected clones were confirmed individually by removing multiple locus-binding clones under standard FISH. Information about the 181 clones is shown in Additional file 1 table S1.

The probes were dissolved in 50% DMSO (400~500 ng/μl density) at 21~23°C and under 40~60% humidity. We used Corning UltraGAPS (Corning, USA) for amine-coated slides, Genemachines OmniGrid 100 (Digilab Genomic Solutions, USA) for DNA spotting, and Telecam SMP4 (Arrayit Corporation, USA) for pin. We followed the general contact type spotter process. Each BAC clone was represented on the array as triplicate spots.

### DNA preparation and labeling for MacArray™ M-chip

Genomic DNAs were extracted using a Gentra Puregen Cell kit (Qiagen Inc., German) from cell lines and clinical samples. The DNAs in 85 of the amniotic fluid samples were directly extracted, while DNAs in nine other cases were extracted after culture. DNA concentration and purity were measured by 1% agarose gel electrophoresis and a NanoDrop ND-100 Full-spectrum UV/Vis Spectrophotometer (Thermo Scientific, USA). Only samples with both 260/280 and 260/230 ratios >1.7 were used for the experiment. The labeling and hybridization protocols described by Pinkel et al. [14] were followed with some modifications, using a Bioprime labeling kit (Invitrogen Carlsberg, CA, USA). Briefly, 21 μl solution containing 500 ng reference DNA or test DNA was mixed with random primers solution and water in a BioPrime Array CGH Labeling System Genomic labeling Module, incubated for 5 min at 95°C, and subsequently cooled on ice. After the addition of 5 μl 10 × dNTPs labeling mixture (1 mM dCTP, 2 mM dATP, 2 mM dGTP, 2 mM dTTP), 3 μl 1 mM Cy-3 or Cy-5 dCTP (GeneChem Inc., Daejeon, Korea), and 40 U Klenow fragment in the BioPrime Array CGH Labeling System Genomic labeling Module, the mixture was gently mixed and incubated overnight at 37°C. Addition of 5 μl Stop Buffer in BioPrime Array CGH Labeling System Genomic labeling Module terminated the reaction. After labeling, unincorporated fluorescent nucleotides were removed using the BioPrime Array CGH Purification Module. In one tube, Cy3-labeled sample DNAs and Cy5-labeled reference DNAs were mixed and 50 μg human Cot-1 DNA (Invitrogen, Carlsberg, CA, USA), 20 μl 3 M sodium acetate and 600 μl cold 100% ethanol were added to precipitate the DNA.

### Array hybridization, imaging and data analysis

The pellet was resuspended in 30 μl hybridization solution containing 50% formamide, 10% dextran sulfate, 2× SSC, 4% SDS and 200 μg yeast tRNA. The hybridization solution was denatured for 10 min at 72°C and subsequently incubated for 1 h at 37°C to block repetitive sequences. Hybridization was performed in slide chambers for 48 h at 37°C. After post-hybridization washes, the arrays were rinsed, spin-dried and scanned into two 16-bit TIFF image files using a GenePix 4200 a two-color fluorescence scanner (MDS, Toronto, Canada). To determine the intensity of each spot, the scanned images were analyzed with MacViewer™ M software (Macrogen, Inc., Korea). The log2-transformed fluorescence ratios were calculated from background-subtracted median intensity values. For each spot, we used 0.25 and -0.25 as thresholds of gain and loss, respectively.

### Fluorescent in situ hybridization (FISH)

Fluorescent in situ hybridization (FISH) was used to verify the microdeletion of Yq11.23, which indicates non-obstructive spermatogenic failure, identified by the MacArray™ M-chip. FISH probes for the region were generated from the human BAC clones RP11-214M24, RP11-140H23, RP11-26B12 and RP11-539D10, purchased from Abbott Laboratories. Bacterial culture and DNA preparations were performed according to the supplier's protocol. Purified BAC clone DNA (1 μg) was labeled with spectrum-red dUTP using a Nick Translation Labeling Kit (Abbott Laboratories, IL). FISH was performed using a standard protocol.

## Results

### MacArray™ M-chip development

We designed a novel, low-density array CGH chip, the MACROGEN MacArray™ M-chip, to detect not only aneuploidies but also micro-level chromosomal aberrations sensitively (see Materials and Methods). Table 2 specifies the characteristics of the 26 diseases (19 micro-deletions and 7 aneuploidies) targeted by the MacArray™ M-chip.

**Table 2:** Target Diseases of MacArray™ M-chip

Type	Disease	Chromosomal Location	Gene/marker
Microdeletion & Duplication	ALAGILLE SYNDROME	20p12	JAG1, MKKS, SHGC-79896
	ANGELMAN SYNDROME	15q11-q13	UBE3A
	CRI-DU-CHAT SYNDROME	5p15.2	TERT
	DIGEORGE SYNDROME	22q11.2	TBX1
	GLYCEROL KINASE DEFICIENCY	Xp21.3-p21.2	GK
	KALLMANN SYNDROME 1	Xp22.3	KAL
	MILLER-DIEKER LISSENCEPHALY SYNDROME	17p13.3	LIS1
	MONOSOMY 1p36 SYNDROME	1p36.33	CDC2L1
	MUSCULAR DYSTROPHY, DUCHENNE TYPE	Xp21.2, 12q21	DMD
	NEUROFIBROMATOSIS, TYPE I	17q11.2	NF1
	NEUROFIBROMATOSIS, TYPE II	22q12.2	NF2
	PRADER-WILLI SYNDROME	15q11-15q13	SNRPN
	SEX-DETERMINING REGION Y	Yp11.3	SRY
	SMITH-MAGENIS SYNDROME	17p11.2	RAI1
	SOTOS SYNDROME	5q35	NSD1
	SPERMATOGENIC FAILURE, NONOBSTRUCTIVE, Y-LINKED	Yq11.23	DAZ
	STEROID SULFATASE DEFICIENCY DISEASE	Xp22.31	-
	WILLIAMS-BEUREN SYNDROME	7q11.2	LIMK1
	WOLF-HIRSCHHORN SYNDROME	4p16.3	WHSC1

Aneuploidy	Trisomy 13	13q12.11~13q33.3	-
	Trisomy 18	18p11.32~18q22.3	-
	Trisomy 21	21q11.2~21q22.3	-
	Abnormal X (X)	Xp11.3~Xq28	-
	Abnormal X (XXY)	Xp11.3~Xq28	-
	Abnormal X (XXX)	Xp11.3~Xq28	-
	Abnormal Y (XYY)	Yp11.2~Yq11.23	-

### Proficiency test using standard reference materials

**Figure 1:**
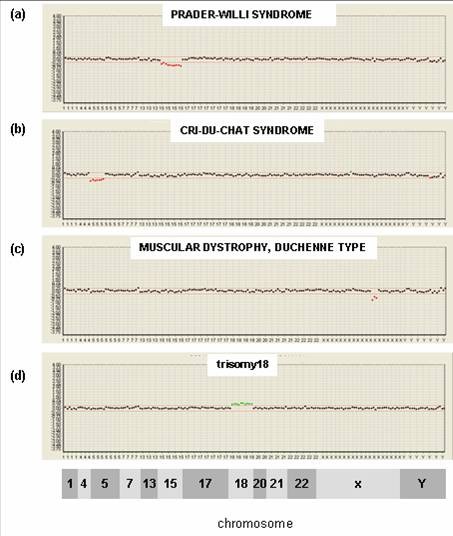
**Detection of chromosomal aberrations by MACROGEN MacArray™ M-chip **. (a) 15q11-15q13 deletion in DNA samples indicating Prader-Willi syndrome was detected by MACROGEN MacArray™ M-chip. The log 2-based test/reference intensity ratios of DNA clones located on chromosome 15 were below -0.25, the threshold indicating chromosomal deletion. (b) Another 5q15.2 deletion in the DNA from cri-du-chat syndrome was detected. (c) An Xp21.2 deletion indicating Duchenne muscular dystrophy was also detected in the same way. An 12q21 deletion reported previously for the same disease was not detected by our platform. (d) Detection of Edward's syndrome (trisomy 18). The aberration was clearly detected by our array system.

We evaluated the feasibility of the MacArray™ M-chip using commercially available standard reference materials. We tested 132 samples bought from Coriell to evaluate the detection of the 15 micro-deletions/duplications and 7 aneuploidies. We successfully detected all these aberrations. Four selected cases detected by the MacArray™ M-chip - 15q11-15q13 micro-deletion causing Prader-Willi syndrome, 5p15.2 micro-deletion causing cri-du-chat syndrome, Xp21.2 micro-deletion causing Duchenne muscular dystrophy and 18p11.32~18q22.3 aneuploidy causing trisomy18 are shown in figure 1. The sample numbers for each target are different from each other, and reference materials for Kallmann syndrome, Sotos syndrome, monosomy 1p36 and the SRY region of Yp were not available. The overall results are presented in Additional file 2 table S2.

### Clinical applications of MacArray™ M-chip

Both cytogenetic analyses and BAC-array CGH were performed for all 94 clinical samples. The overall results are shown in Additional file 3 table S3. One case showing the 46,XX normal female karyotype failed in the MacArray™ M-chip test because of poor hybridization. Genomic aberrations were detected in 20 cases (Table 3). Three balanced rearrangements were identified in only conventional karyotyping. These balanced rearrangements were two cases of inv[9], which was considered a normal variation, and one case of translocation, t[8,11].

**Table 3:** Abnormal cases detected by conventional karyotype analysis and MacArray™ M-chip test.

sample No.	Karyotype analysis	MacArray™ M-chip Test
S008	46,XY,inv(9) (q12q13)	arr(1,4,5,7,13,15,17,18,20-22)x2,(XY)x1
S026	46,XY	arr Yq11.223(23611993-25573091)x0
S031	46,XY	arr Yq11.223(23611993-25573091)x0
S035	46,XX, inv(9)(q12q13)	arr(1,4,5,7,13,15,17,18,20-22,X)x2
S044	46,XY,t[8;11](p21;p15.5)	arr(1,4,5,7,13,15,17,18,20-22)x2,(XY)x1
S048	47,XX,+21	arr(21)x3
S049	47,XX,+18	arr(18)x3
S052	47,XY,+21	arr(21)x3
S055	47,XY,+21	arr(21x3)
S061	47,XY,+21	arr(21)x3,Yq11.223(23611993-25573091)x0
S065	47, XX,+21	arr(21x3)
S068	46, XY	arr Yq11.223(23611993-25573091)x0
S069	46,XY	arr Yq11.223(23611993-25573091)x0
S070	47,XY,+21	arr(21)x3
S073	47,XXY	arr(X)x2,(Y)x1
S074	46,XY	arr Yq11.223(23611993-25573091)x0
S075	46,XY	arr Yq11.223(23611993-25573091)x0
S083	47,XY,+21	arr(21)x3
S086	47,XY,+13	arr(13)x2,Yq11.223(23611993-25573091)x0
S087	47,XXY	arr(X)x2,(Y)x1

**Figure 2:**
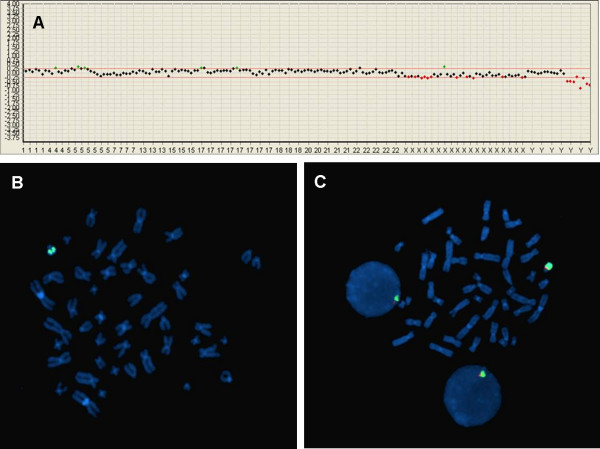
**Confirmation of Yq12 deletion by FISH **. (a) The MacArray™ M-chip showed deletions in the Yq region. (b). In a patient with Yq deletion, the WCP Y signal (green) was detected but the DAZ locus signal (orange) was absent. (c). Normal male control for Yq12 deletion. The signal for the DAZ locus was detected in both chromosome and nucleus.

On the other hand, we identified a microdeletion of the Yq11.23 region harboring the DAZ gene by MacArray™ M-chip in eight cases, seven showing normal male karyotype and one showing trisomy 13. All eight of these cases were confirmed by FISH (Figure 2). We detected no other microdeletions in our series.

## Discussion

We have developed a targeted low-density BAC-array DNA chip, the MacArray™ M-chip, as a adjunctive prenatal genetic test to conventional karyotyping for simultaneous prenatal screening of chromosomal abnormalities and microdeletion syndromes in South Korea.

The greatest benefit of aCGH is its ability to detect a very small genomic imbalance that cannot be detected by conventional G-banded karyotyping. It is also a high-throughput method, detecting hundreds or thousands of discrete loci in a single simultaneous assay, in contrast to FISH and QF-PCR, which have been used for aneuploidy screening because they give rapid results but only detect a few loci a time. A microarray DNA chip can be constructed using various-sized targets ranging from oligonucleotides (25-85 bp) to BACs (80-200 kb) [15]. Microarrays constructed using a high-resolution and whole-genome approach can identify very many polymorphisms with not only pathogenic deletions/duplications but also uncharacterized genomic imbalances. About two thirds of genomic imbalances detected by prenatal array CGH have been interpreted as probably benign and of no clinical significance [12].

In contrast to genome-wide aCGH chips, this targeted BAC aCGH chip contains clones only for regions with known clinical significance. While it had lower overall detection rates than genome-wide arrays, it greatly reduced the number of benign CNVs and other anomalies of unclear clinical significance that are otherwise detected [15]. Shaffer et al. [16] reported that 5.6% of cases showed clinically relevant abnormalities, with only 2.4% and 0.9% showing familial variants and unclear significance, respectively. A targeted BAC array might therefore be considered more applicable to clinical diagnosis.

Application of arrays to prenatal diagnosis requires great care because even the detection of a familial variant raises maternal anxiety. Therefore, ruling out insignificant CNVs and unclear results is critical. The MacArray™ M-chip is specially designed for prenatal screening of chromosomal abnormalities that should be detected in the second trimester of pregnancy. Because the array only contains 181 clones with characterized clinical relevance, we greatly reduced the uncertainty of results and also the cost burden on the patients. In this study, we readily detected 15 microdeletions and 7 aneuploidies in 132 reference materials purchased from the Coriell Cell Resource. However, we identified no CNVs or inconclusive alterations in any of those 132 reference materials (Additional file 2 table S2). All chromosomal abnormalities found by conventional cytogenetic analysis were also detected by the MacArray™ M-chip except for the apparently balanced structural abnormalities inv[9] and t[8,11]. The MacArray™ M-chip could not identify aneuploidies of chromosomes 2, 3, 6, 8, 9, 10, 11, 12, 14, 16 and 19, which are rarely detected in amniocentesis. However, Additional clones for those chromosomes will be added in the next upgraded platform. Our platform also could not be used to detect triploidy. The array CGH platform is not appropriate for detecting polyploidy because it is difficult to separate polyploidy from systematic error (dye bias)

On the other hand, in eight prenatal cases, the MacArray™ M-chip successfully identified the microdeletion on the long arm of Y chromosome (Yq11) that is considered to be associated with non-obstructive spermatogenic failure. Because this is a prenatal diagnostic chip, our purpose is not to detect the DAZ microdeletion prenatally. The DAZ1 probe represents Yq and was used to identify Y chromosome aneuploidies such as 47,XXY.

Deletions in the Yq11 region are hard to distinguish from normal variations in routine cytogenetic analysis and are usually detected by polymerase chain reaction (PCR) using short tandem repeat (STR) markers. Four of the eight microdeletions were inherited from their fathers and the fetuses were fertilized by intracytoplasmic sperm injection (ICSI). The deletions in the fathers were identified by PCR prior to in vitro fertilization procedure (IVF). We did not perform a paternal Y deletion test in the other four cases, so we did not know whether the deletion was inherited. As shown in figure 2, deletions of Yq11 were confirmed by FISH in all eight cases. In this clinical application, we could detect only DAZ microdeletions in addition to anueploidies.

It would be better to extend this study to many more samples to explore the incidence of microdeletions and duplications for the given indications further. We plan to revise and upgrade the chip so that it will contain more BAC clones for microdeletion syndromes and subtelomeric regions for each chromosome. Of course, we will apply the chip to many more samples and extend its usage, and hence be able to discuss the benefits of array CGH screening in comparison to FISH and QF-PCR.

## Conclusions

We have successfully designed and applied a BAC-based array CGH platform for prenatal diagnosis. Even though the MacArray™ M-chip contains a very limited number of clones, this system can provide a highly efficient and accurate method for the prenatal screening of chromosome aneuploidies and microdeletions, making it a clinically useful addition to conventional G-banded karyotyping.

## Competing interests

Grant sponsor: Korea Health 21 R&D Project, Ministry of Health & Welfare, Republic of Korea; Grant number: A050558

## Authors' contributions

JHP participated in the design of the study, wrote and given final approval of the version to be submission. JHW integrated and analyzed array CGH data and wrote the manuscript. SHS analyzed and interpreted data and carried out drafting of the manuscript. SJY conceived and designed the study. YMC conceived and designed the study. KSY conceived and designed the study and supervised the project. DHC conceived and designed the study and carried out critical revision of the manuscript for important intellectual content and supervised the project.

All authors read and approved the manuscript.

## Pre-publication history

The pre-publication history for this paper can be accessed here:

http://www.biomedcentral.com/1471-2350/11/102/prepub

## Supplementary Material

Additional file 1 Table S1. Contents of MACROGEN MacArray™ M-chip Click here for file

Additional file 2 **Table S2. Performance of MacArray™ M-chip in detecting 26 diseases **. Click here for file

Additional file 3 **Table S3. Overall results of clinical applications using MacArray™ M-chip and karyotype analysis to detect chromosomal abnormalities **. Click here for file
